# Optimization of physical and dielectric properties of Co-doped ZnO nanoparticles for low-frequency devices

**DOI:** 10.1371/journal.pone.0287322

**Published:** 2023-11-22

**Authors:** Adil Muhammad, Muhammad Sajid, Muhammad Nouman Khan, Muhammed Sheraz, Awais Khalid, Pervaiz Ahmad, Satam Alotibi, Hamed M. Al-saidi, Nebras Sobahi, Md Mottahir Alam, Sultan Althahban, Ahmad M. Saeedi, Hasan B. Albargi

**Affiliations:** 1 Department of Physics, Islamic International University, Islamabad, Pakistan; 2 School of Material Science, Beijing Institute of Technology, Beijing, China; 3 School of Optics and Photonics, Beijing Institute of Technology, Beijing, China; 4 Department of Physics, Govt: Post Graduate College Mardan, Mardan, Pakistan; 5 Department of Physics, Hazara University Mansehra, Khyber Pakhtunkhwa, Pakistan; 6 Department of Physics, College of Science and Humanities in Al-Kharj, Prince Sattam bin Abdulaziz University, Al-Kharj, Saudi Arabia; 7 Department of Physics, University of Azad Jammu and Kashmir, Muzaffarabad, Pakistan; 8 Department of Chemistry, University College in Al-Jamoum, Umm Al-Qura University, Makkah, Saudi Arabia; 9 Department of Electrical and Computer Engineering, King Abdulaziz University, Jeddah, Saudi Arabia; 10 Department of Mechanical Engineering, Jazan University, Jazan, Saudi Arabia; 11 Faculty of Applied Science, Department of Physics, Umm AL-Qura University, Makkah, Saudi Arabia; 12 Faculty of Science and Arts, Department of Physics, Najran University, Najran, Kingdom of Saudi Arabia; 13 Promising Centre for Sensors and Electronic Devices (PCSED), Najran University, Najran, Kingdom of Saudi Arabia; National Research Centre, EGYPT

## Abstract

In this study, zinc-oxide (ZnO) nanoparticles (NPs) doped with cobalt (Co) were synthesized using a simple coprecipitation technique. The concentration of Co was varied to investigate its effect on the structural, morphological, optical, and dielectric properties of the NPs. X-ray diffraction (XRD) analysis confirmed the hexagonal wurtzite structure of both undoped and Co-doped ZnO-NPs. Scanning electron microscopy (SEM) was used to examine the morphology of the synthesized NPs, while energy-dispersive X-ray spectroscopy (EDX) was used to verify their purity. The band gap of the NPs was evaluated using UV-visible spectroscopy, which revealed a decrease in the energy gap as the concentration of Co2+ increased in the ZnO matrix. The dielectric constants and AC conductivity of the NPs were measured using an LCR meter. The dielectric constant of the Co-doped ZnO-NPs continuously increased from 4.0 × 10^−9^ to 2.25 × 10^−8^, while the dielectric loss decreased from 4.0 × 10^−8^ to 1.7 × 10^−7^ as the Co content increased from 0.01 to 0.07%. The a.c. conductivity also increased with increasing applied frequency. The findings suggest that the synthesized Co-doped ZnO-NPs possess enhanced dielectric properties and reduced energy gap, making them promising candidates for low-frequency devices such as UV photodetectors, optoelectronics, and spintronics applications. The use of a cost-effective and scalable synthesis method, coupled with detailed material characterization, makes this work significant in the field of nanomaterials and device engineering.

## 1 Introduction

Metal oxides are thought to be promising materials because of their unique properties such as quantum size effects, large aspect ratios, chemical and thermal stabilities, and so on [1–4]. Among these, Zinc oxide (ZnO) is the wide band gap (3.37 eV) inorganic compound with large excitation energy (60 m eV) belonging to II-VI group. It is usually found in the following basic crystal structures, i.e., wurtzite, Zinc blend, and rock salt [5]. The hexagonal wurtzite structure of ZnO is stable at room temperature and zinc blend structure can be found in a metastable state. Hetro-epitaxial growth on a cubic symmetric substrate can stabilize the zinc blend structure. The lattice parameters of ZnO’s wurtzite structure are a = 3.24 Å and c = 5.20 Å [6,7]. As a result of its appropriate physical and chemical properties, ZnO is well-suited for the design of magnetic, electrical and optical devices such as transistors, ZnO-based LEDs and photosensors; solar cells; optical switches; photocatalysts and piezoelectric transducers [8,9]. Furthermore, ZnO has the potential to be very useful in biomedical applications, antireflection coating, and transparent electrodes in solar cells. Applications are restricted by nanoscale high resistivity and high electron-hole pair recombination rate despite its excellent properties [10,11]. Size, morphology tuning and doping with suitable element like TiO_2_, CuO, and SiO_2_ are the alternative approaches that make them usual for various applications [12]. Various shapes of ZnO nanostructures such as nanorods, nanoparticles, nanocubes, nanowires and nano flowers are already reported [13]. Transition metals (TM = Co, Mn, Fe, Ni etc.) are the most promising materials used to tune their electrical, optical and magnetic properties [14]. Among them, Co is considered as the most effective element for tailoring the optical and dielectric properties due its compatibility with ZnO at nanoscale [15].

The optical and transport characteristics of semiconductor crystal structures are primarily influenced by intrinsic and fundamental flaws. Due to the larger band gap of ZnO than that of GaN, its optical properties are very interesting to researchers [16]. The energy band gap of the composite is altered depending on the amount of doping when TM (Fe, Co, Ni, Mn, etc.) are doped into ZnO.

The band electrons of transition metal ions and exchange interaction of s-pd between confined d state electrons of ZnO semiconductor could be the reason behind smaller energy band gap. The BM (Bursteion Moss) theory says that electrons from dopant elements can live at the bottom of the conduction band, which could explain why transition metals doped into ZnO made the band gap bigger [17,18]. According to the literature review, there is an increase in the crystallite size of ZnO nanoparticles as the dopant ratio of Co ion in ZnO increases. We can calculate the average size of nanoparticle crystallites using Scherrer’s formula and Williamson Hall analysis [19–21]. When the frequency is raised, both pure and Co doped ZnO-NPs have lower dielectric constants and higher a.c. conductivities. On the bases of Maxwell and Weigner modes Koop’s theory the frequency is explained in which dielectric constant drops [22]. To synthesize the TM doped ZnO nanoparticles different approaches including evaporation condensation, solgel, hydrothermal, sputtering, and co-precipitation can be used [23–26]. These NPs can then be used in a variety of applications. However, in our study, we used the co-precipitation technique to produce pure and Co-doped ZnO-NPs (Zn_1-x_Co_x_O) because it is simple and less expensive than the other methods. It is the ultimate goal of this study to use the co-precipitation technique to prepare controllable preparations of Co^2+^ substitute ZnO-NPs, to better understand how the d block element Co^2+^ can be used to dope ZnO in order to enhance its significant properties for the development of novel applications such as spintronics, solar cells photonic devices and Ultraviolet photo sensers, as well as to provide optimized materials for thermoelectric power generators (TEG).

While several studies have examined the magnetic, biomedical, and photocatalytic properties of Co-doped ZnO nanoparticles, there has been limited research on their dielectric properties [27,28]. This study focuses on the synthesis of Co-doped ZnO nanoparticles using a simple coprecipitation method with varying Co concentrations, followed by a comprehensive analysis of their structural, optical, and dielectric properties using XRD, UV-visible spectroscopy, FTIR, SEM, and dielectric spectroscopy techniques. The results demonstrate that Co doping significantly affects the dielectric properties of ZnO nanostructures, highlighting the potential of these materials for applications in UV photodetectors, optoelectronics, and spintronics devices. This work presents a novel contribution to the field of nanomaterials by providing a deeper understanding of the dielectric properties of Co-doped ZnO nanoparticles synthesized using a cost-effective and scalable technique.

## 2 Materials and methods

The analytical grade reagents used as raw materials were procured from Sigma-Aldrich. Without further purification, these chemicals were subsequently employed these reagents were subsequently employed for the synthesis of un-doped and Co-doped ZnO NPs. Nanomaterial was synthesized via a simple chemical process using Zinc acetate dehydrate [Zn (CH_3_COOH)_2_.2H_2_O, 99%] as a starting material, Cobalt acetate tetra hydrate [Co (CH_3_COOH)_2_.4H_2_O, 99%] as a dopant material, sodium hydroxide (NaOH, 99%) to adjust pH value. While acetic acid (CH3COOH, 99.9%) and distilled water (H_2_O) were used as a surfactant and reaction medium respectively.

### 2.1 Synthesis of ZnO and Co-Doped ZnO-NPs

A required amount of [Co (CH3COOH)_2_.4H2O] and [Zn (CH3COOH)_2_.2H2O] was obtained in a typical synthesis by adding the precursors in 100 ml of distilled water with continuous magnetic stirring. NaOH solution was added drop wise to increase its pH value. The reaction was then aged for 1h at 100° C and adjust the pH up to 11 under vigorous magnetic stirring. The precipitant was collected at room temperature after the reaction was completed and washed with ethanol and distilled water (1:1) using a centrifuge. Finally, the obtained black powder was dried overnight in an electric oven set to 100° C. The dried powder was then annealed at 450° C for 3 hours to increase the crystallinity. Similarly, all other samples were prepared using the same procedure but with different Co dopant concentrations. Throughout the preparation of these samples, the following chemical reaction has occurred.


$$(1‐x)\;Zn\;{\left(CH_{3}COOH\right)}_{2}.2H_{2}O+x\;Co{\left(CH_{3}COOH\right)}_{2}.4H_{2}OZn_{1‐x}Co_{x}O+6H_{2}O+3CO_{2}+5C$$ (1)

### 2.2. Characterization

X-Ray Diffractometry (XRD) CuKa (Model: JDX-3532, JEOL, Japan) was used for structural investigations of prepared NPs. Scanning Electron Microscope (SEM) (Model JSM6410, JEOL, Japan) was employed to study the morphological aspects of the prepared pure and Co doped ZnO-NPs. Ultraviolet-Visible Spectroscopy (UV-Spectroscopy) by Perkin-Elmer (Lambda 25-UV) and Inductor, Capacitor and resistor meter (LCR-meter) (E4980A) were used to study the optical (band gap) and dielectric properties of the prepared NPs.

## 3. Results and discussion

### 3.1. X-Ray Diffractometry (XRD) analysis

**Fig 1:**
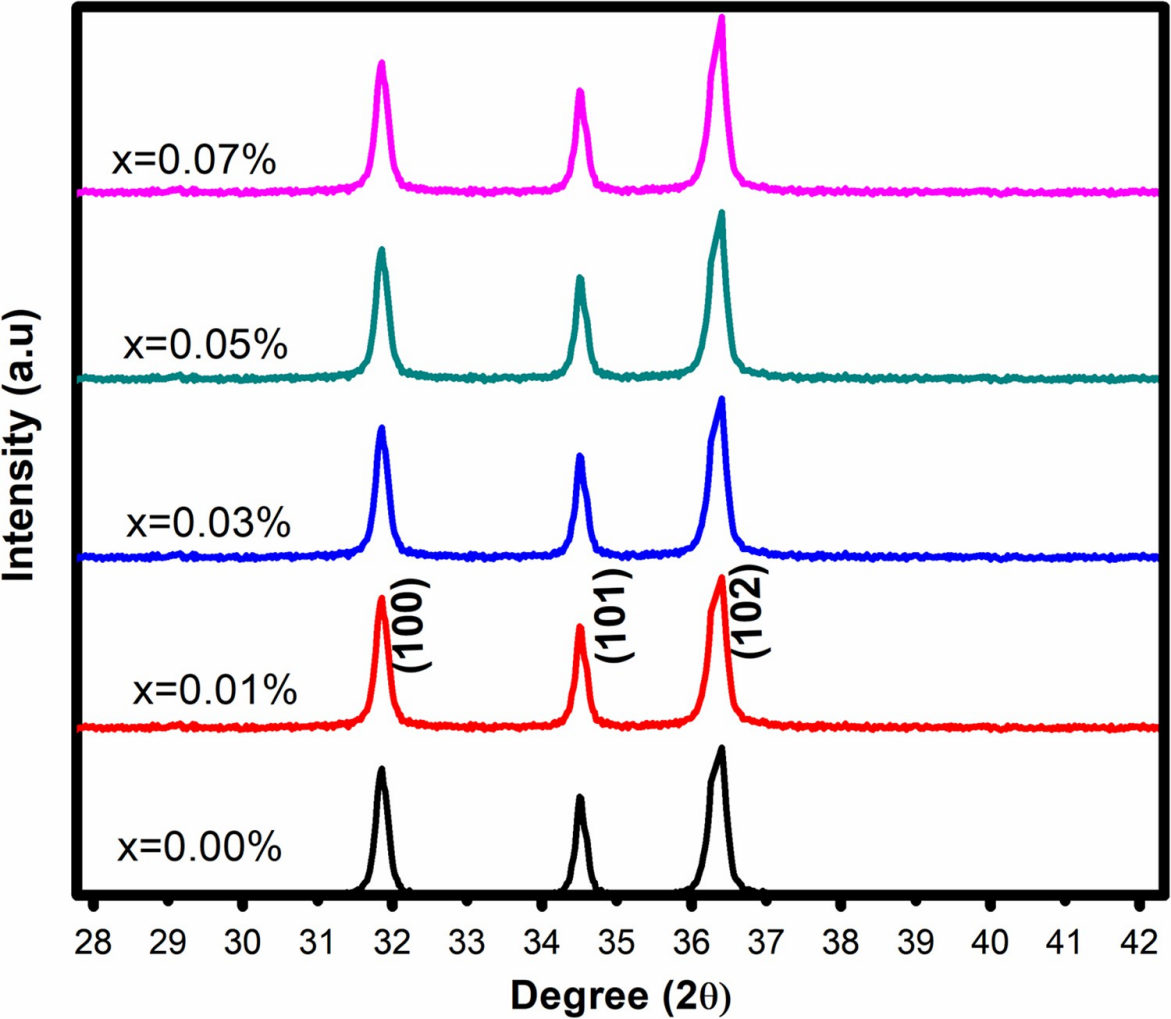
XRD pattern of as synthesized NPs.

The structural behaviour of pure and Co doped ZnO nanoparticles was studied using a series of controlled experiments. The crystallinity of the prepared NPs was examined using XRD. An XRD pattern with the typical hexagonal wurtzite structure of ZnO is shown in Fig 1 (JCPDS card no: 036–141). The bands represent the various well observed peaks associated with (100), (101), and (102) that agree to wurtzite ZnO at 32°, 34.5°, and 36.5°, respectively. The intensity of the (101) peak increased slightly with increasing Co percentage, indicating an increase in crystallite size and the absence of impure phases of other oxides regardless of dopant concentration.

From the stronger XRD peak, the Scherer’s equation is used to determine the crystallite size of all prepared nanoparticles (101).


$$D_{hkl}=\frac{0.9\lambda }{\beta \cos\theta }$$ (2)

In which "D" stands for crystallite size, "β" is the entire width at half maximum of the diffraction peak, " λ " for the incoming X-ray wavelength (Cu k_α_ = 1.54056 Å), and angle of diffraction is represented by "θ".

In X-ray diffraction (XRD), the lattice parameters of a crystal can be calculated using the Bragg equation:

nλ=2dsinθ (3)

where n is the order of the reflection (usually 1), λ is the wavelength of the X-rays used, d is the spacing between crystal planes, and θ is the Bragg angle (the angle between the incident X-ray beam and the crystal plane that reflects the X-ray beam).

**Fig 2:**
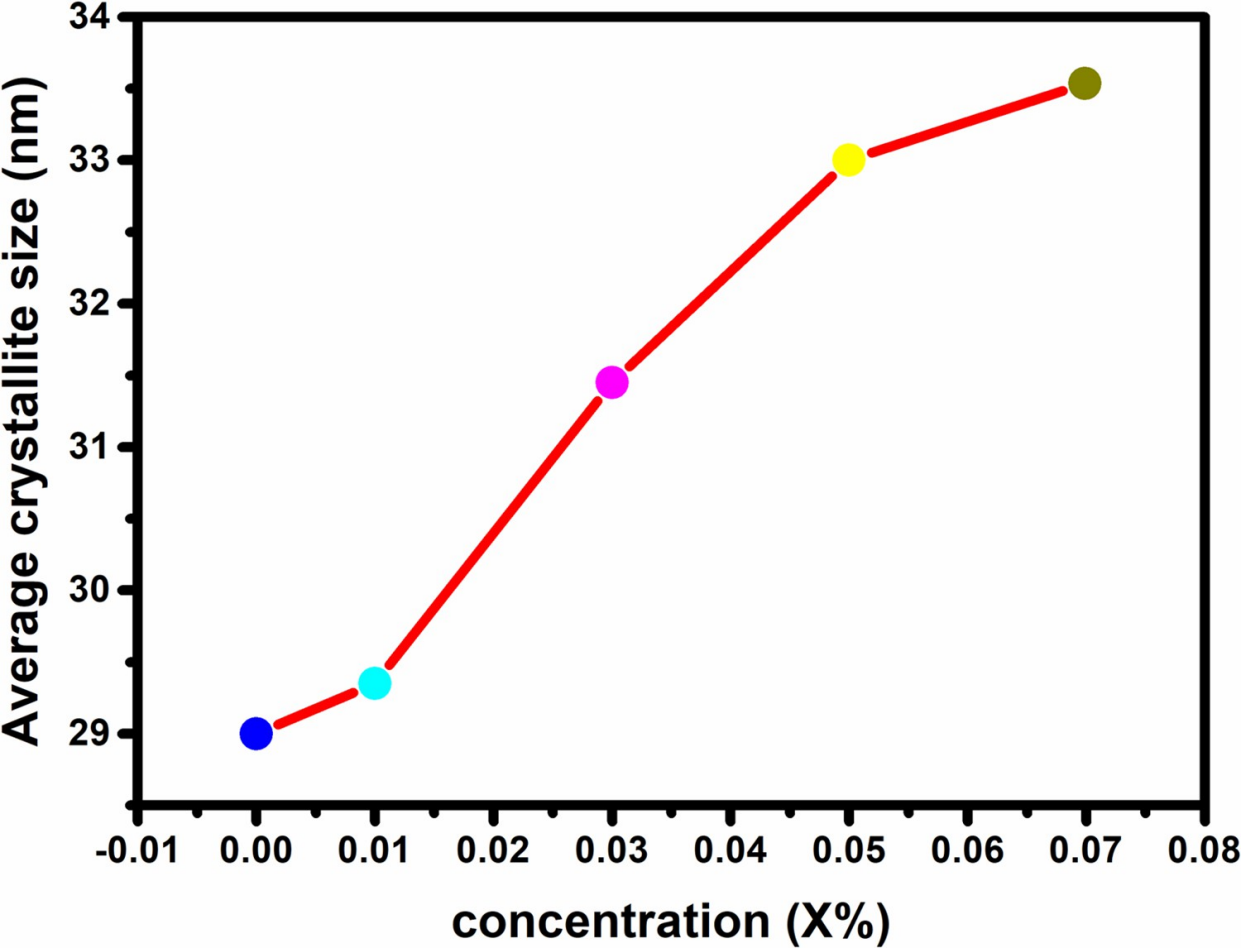
Co dopant Concentration vs. average crystallite size.

**Fig 3:**
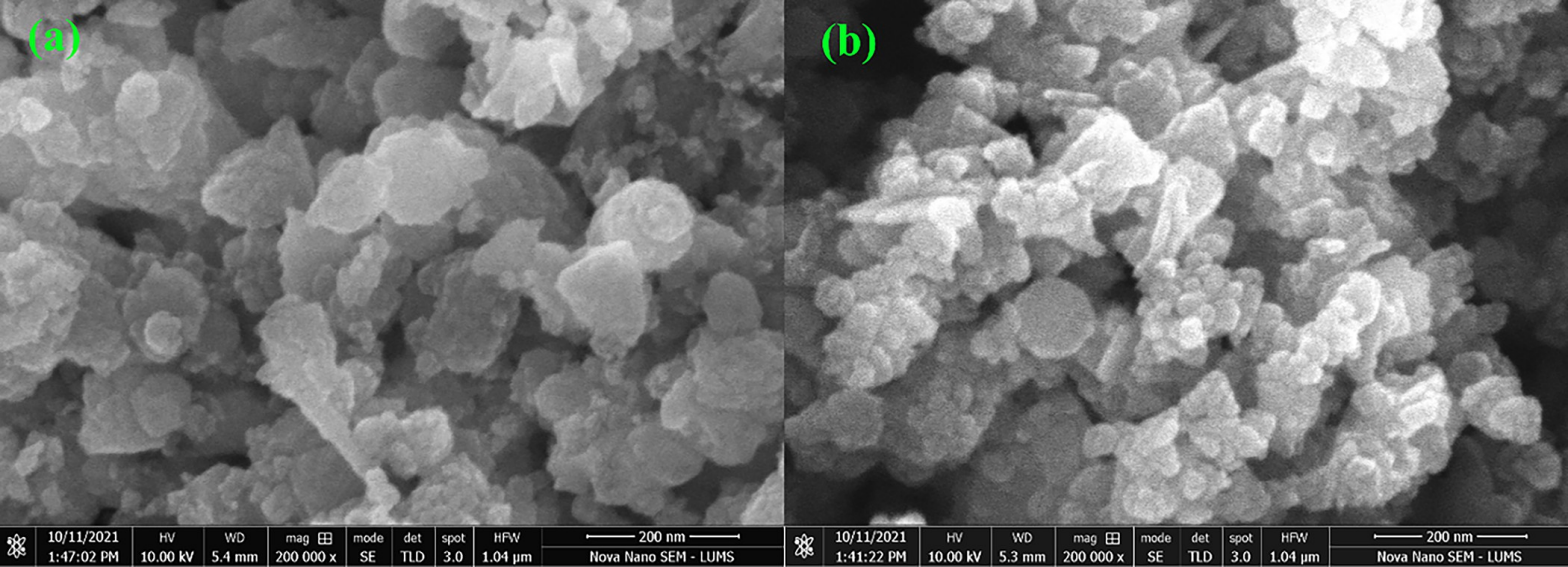
(a) Display a SEM image of pure ZnO-NPs. **(b)** Display a SEM image of 0.05% Co doped ZnO-NPs.

The sharp intense peaks (101) for all dopant concentrations including 0.00%, 0.01%, 0.03%, 0.05%, and 0.07%, which, were used to compute the crystallite size of all produced samples i.e., 29 nm, 31 nm, 31.45 nm, 33 nm, and 33.5 nm respectively, as shown in Figs 2 and 3 demonstrates that crystallite size grows with increasing Co percentage. The replacement of neighboring Zn with other Co atoms induced lattice assembly distortion due to which rise in crystallite size with increasing Co^+2^ dopant content in ZnO matrix is driven by Co beginning to occupy interstitial locations in addition to substitutional positions. This performance could be attributable to the difference in slit radii between divalent Zn (0.60 Å) and divalent Co (0.58 Å) in tetrahedral coordination. The increase in crystallite size reveals the presence of cobalt in the ZnO lattice. During cobalt doping, distortion is caused by the dopant atoms due to a mismatch between ionic radii of Zn^2+^ and Co^2+^. This mismatch led to deformation at various points in the ZnO matrix. The increase in deformations results in enhancement in the average crystallite size at higher concentrations of cobalt. As a result, higher cobalt concentrations caused interstitial atoms to be present, which contributed to increase the lattice volume and average crystal size.

The values of lattice parameters a, b and c for pure and Co doped ZnO-NPs are calculated using the formula in Table 1.

**Table 1:** Lattice parameter formula.

Samples	a = b (nm)	C (nm)
ZnO	0.3248	0.5132
Zn_0.99_Co_0.01_O	0.3248	0.5193
Zn_0.97_Co_0.03_O	0.3249	0.5196
Zn_0.95_Co_0.05_O	0.3250	0.5204
Zn_0.93_Co_0.07_O	0.3252	0.5211

### 3.2 Scanning electron microscopy

Using a SEM, the sample’s morphology of the synthesized ZnO and Co doped ZnO-NPs was examined. SEM has opened doors in a variety of disciplines, from engineering to chemistry, giving researchers new and beneficial knowledge about microscopic processes with macroscopic repercussions [29]. Fig 3A and 3B shows SEM images of nanoparticles of pure zinc oxide (Zn_1-x_Co_x_O) and nanoparticles with Co^+2^ added (Zn_1-x_Co_x_O) with X = 0.00 and 0.05). Pure and Co-doped ZnO (Zn_1-x_Co_x_O) are spherical in shape. The average particle size of pure ZnO and Co doped ZnO-NPs is 87 nm and 45 nm which is observed using Image J software, which supports the XRD analysis’s assertion even more.

### 3.3 Energy dispersive X-ray spectroscopy

**Fig 4:**
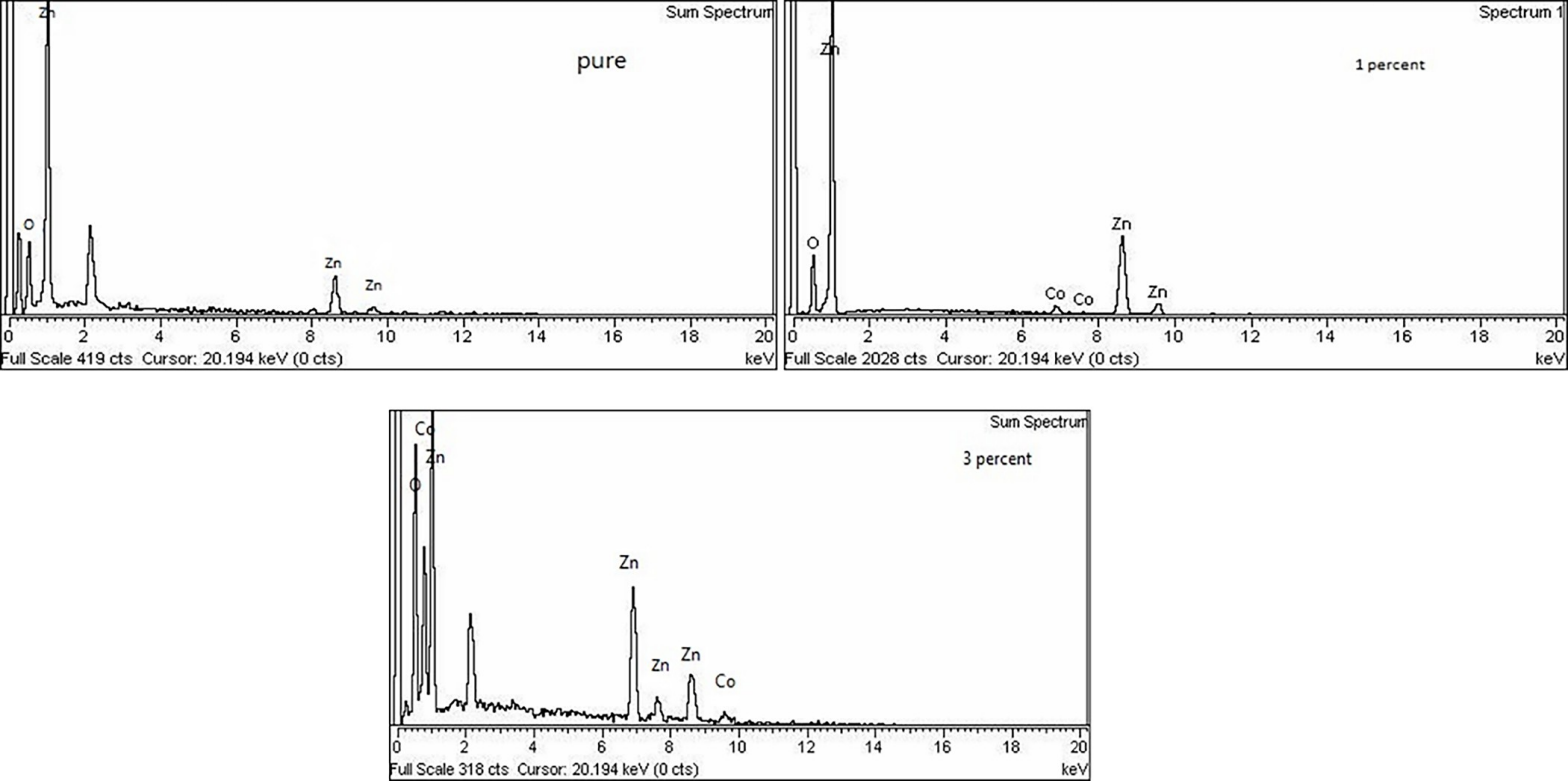
(**a**): EDX spectrum of an undoped sample. (b) Display the EDX spectrum of a Zn_0.99_ Co_0.01_ O sample. (c): Display the EDX spectrum of a Zn_0.97_ Co_0.03_ O sample.

The EDX spectra of pure zinc oxide nanoparticles (a), Co doped nanoparticles (b), and Co doped nanoparticles (c) with doping values of 0.00 to 0.01 are shown in Fig 4. Nanoparticles of pure zinc oxide (ZnO) show only the zinc (Zn) and oxygen (O) peaks in Fig 4(A). Peaks in the Zn, O, and Co elements are shown in Fig 4(B) and 4(C), with the Co quantity increasing, indicating that Co^+2^ is effectively substituted within the ZnO matrix. No other peaks can be seen other than C (carbon) and Ag (gold) metals that were used in coating to cause scattering back of electrons from the materials during SEM. As expected, the EDX peaks showed that cleaned samples were pure. The percentage of Zn, O and Co different samples are shown in Table 2.

**Table 2:** Shows the percentage of Zn, O and Co different samples.

Samples	Zn (W%)	O(W %)	Co (W%)
**ZnO**	53.87	46.13	0
**Zn_0.99_Co_0.01_O**	49.69	49.09	0.22
**Zn_0.97_Co_0.03_O**	45.24	50.33	4.43
**Zn_0.95_Co_0.05_O**	42.27	51.87	5.86
**Zn_0.93_Co_0.07_O**	37.92	54.31	7.77

### 3.4 Energy band gap measurement

Investigating the optical properties of undoped and doped ZnO NPs, as well as other semiconducting NPs, is a common use of UV-visible absorption spectroscopy. By using the Tauc relationship as shown below, the optical band gap of cobalt-doped zinc oxide (Zn_1-x_Co_x_O) and pure zinc oxide was measured.


$${\left(ɑh\upsilon \right)}^{2}=A{\left(h\upsilon ‐E_{g}\right)}^{n}$$ (4)

**Fig 5:**
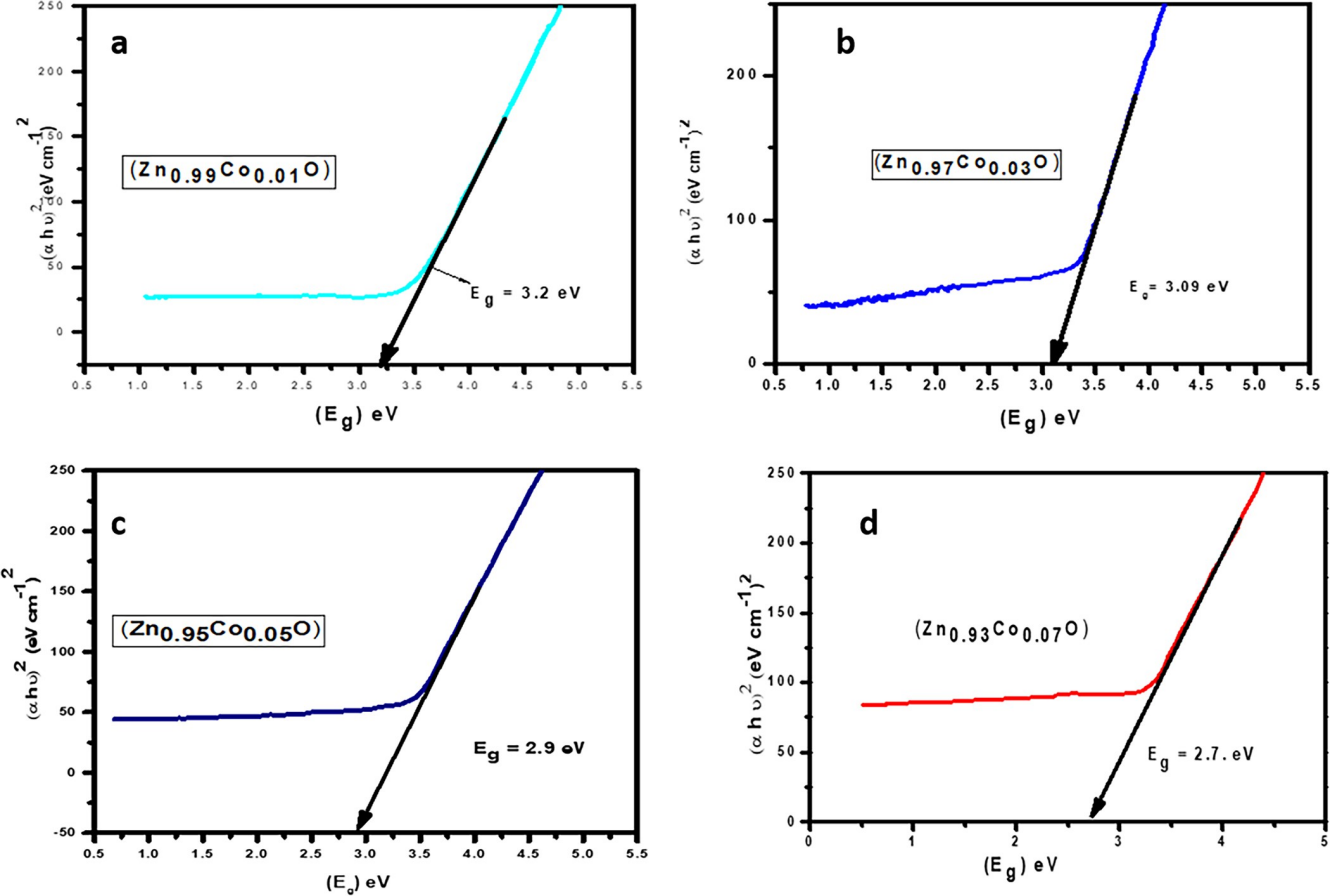
Variation in band gap energy of (a) 0.01%, (b) 0.03%, (c) 0.05%, and (d) 0.07% Co doped ZnO-NPs.

In the above expression, h represents Planck’s constant, ν signifies the photon energy, A represents a constant, and the optical band gap is denoted by E_g_; "ɑ" represents the absorption coefficient (ɑ = $2.303\frac{A}{t}$) where "A" and "t" represents the absorbance and thickness of the sample. The absorption value of a direct band gap semiconductor is n = $\frac{1}{2},\frac{3}{2}$, 2 *or* 3, conditional to the electronic transition’s nature which were responsible for the absorption. ZnO is a semiconductor with a straight band gap, hence n = $\frac{1}{2}$ is its characteristic value. By plotting (ɑhʋ)^2^ against photon energy (h), we can calculate the E_g_ of undoped and varied concentrations of Co doped ZnO NPs, as reported in Fig 5 and Table 3.

**Table 3:** Energy band gap of various dopant concentration of Co-doped ZnO-NPs.

Samples	Eg (eV)
ZnO	3.4 eV
Zn0.99Co0.01O	3.2 eV
Zn0.97Co0.03O	3.09 eV
Zn0.95Co0.05O	2.9 eV
Zn0.93Co0.07O	2.7 eV

**Fig 6:**
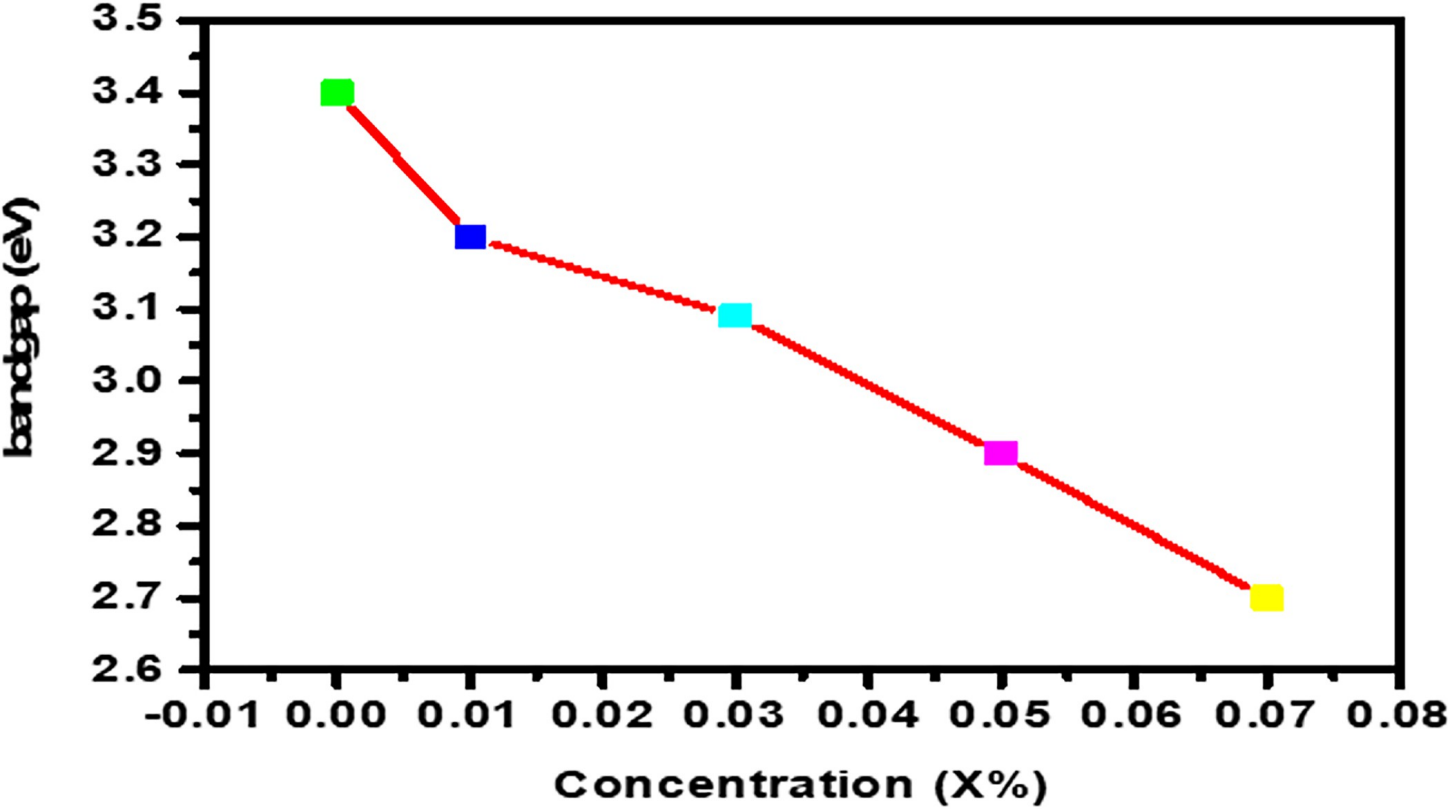
Variation in band gap energy with respect to Co dopant concentration in ZnO-NPs.

It has been discovered that the band gap energy of ZnO reduces as Co^2+^ ion dopant concentration rises. The band electrons of Co^2+^ ions, which replace Zn^2+^ ions and exchange interaction of sp-d between confined "d" electrons may be responsible for potentially lower band gap energy. When doping Co into ZnO nanoparticles, however, Sajid Ali et al. observed an increase in the band gap width with the escalating intensity of doping as shown in Fig 6. It could be due to a drop in the lattice parameter.

### 3.5 Dielectric properties

These diluted oxides magnetize semiconducting nanoparticles dependent on dielectric properties which were influenced by a variety of factors, including the ratio of Zn^+2^ to Co^+2^ ions, annealing temperature manufacturing method, chemical composition, and size of particle, and. An important property of Co doped ZnO-NPs is their dielectric properties, which can be used in a variety of applications including optoelectronics, recording media, microwave devices, and transport properties. To investigate the dielectric characteristics, LCR meter has been employed to the synthesized NPs. The sample in a dry powdered form were taken and converted into pallets form before the dielectric measurement. The measurements were carried out in the 1 KHz to 2 MHz frequency range at room temperature in the IIUI’s nanoscience lab.

#### 3.5.1 Dielectric constant

**Fig 7:**
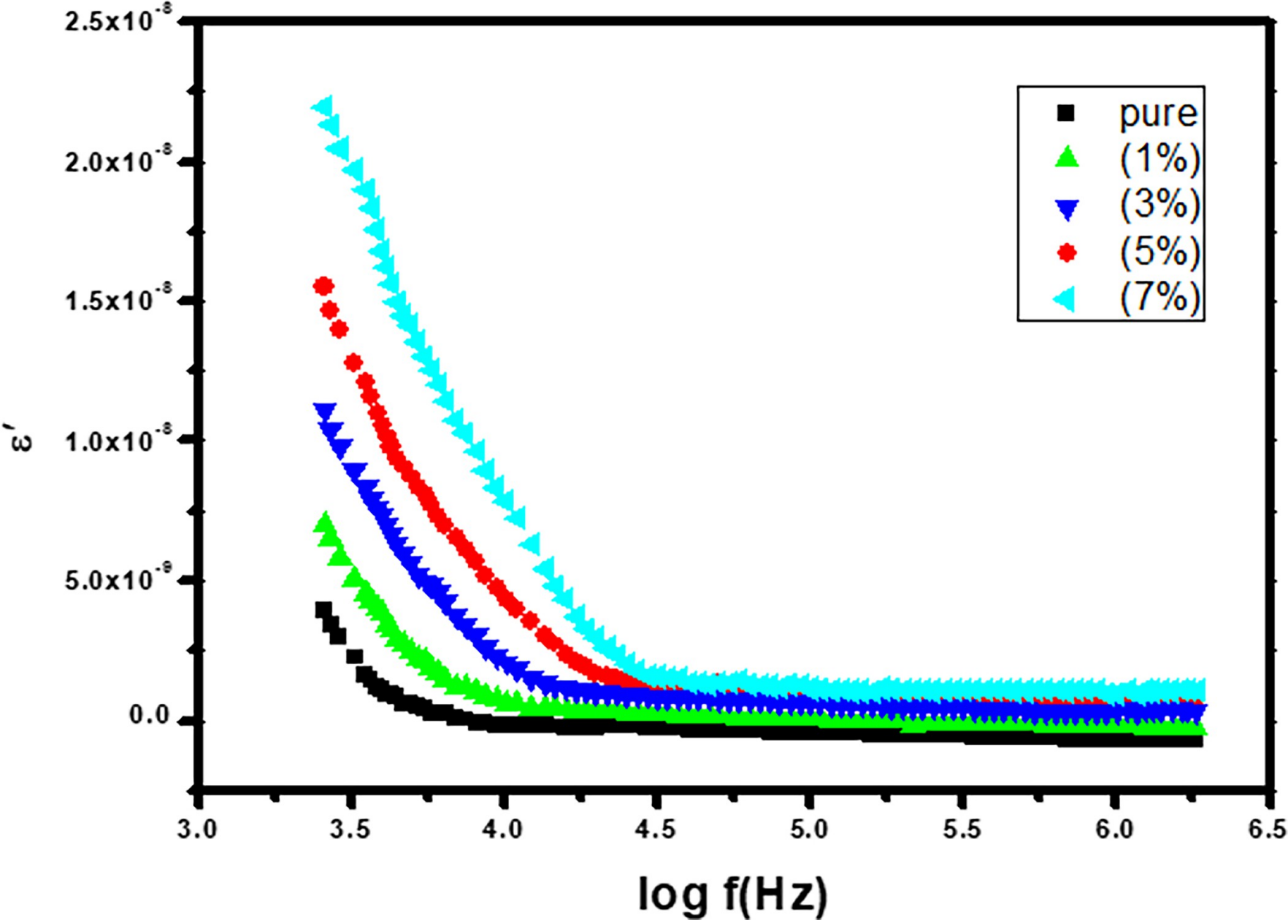
Show real part of dielectric constant with varying frequency.

The Co_-_doped ZnO-NPs with varying dopant concentrations are shown in Fig 7 along with their dielectric constant (**ε**^**’**^). Every sample’s dielectric constant (**ε**^**’**^) has been found to be maximum at low frequencies, and it exponentially decreases as frequency increases. The polarization mechanism with an external field is responsible for this phenomenon. Maxwell-Wagner model and the Koop’s phenomenological theory have both been used to explain the dielectric actions of synthesized nanoparticles [30–32].

Koop postulated that crystals have two layers: grains and grain boundaries, with the former serving as a good conductor at low frequencies and the latter being a poor conductor. When these samples are exposed to an external electric field, electrons diffuse to the grain boundaries. Since grain boundaries are very resistive, this causes the electrons to accumulate and produce polarization. This process is known as hooping. At higher frequencies, the dielectric constant drops dramatically as the dipole moment does not coincide with the applied field, and the field has to "duck" to get to the grain boundary electrons [33].

The Maxwell-Wagner model, which has two layers but a uniform medium, is a reference to Koop’s phenomenological theory. Since space charge polarization causes a very high gathering of charges at grain boundaries at low frequencies, each sample’s dielectric values are very high for this frequency. Every sample’s dielectric constant value decrease as the applied electric field frequency increases, but as the frequency increases further, a point is reached where space charge is not involved in the polarization phenomenon, demonstrating that polarization is independent of the applied electric field at high frequencies [34]. The doping level concentration has an impact on the dielectric constant of synthesized nanoparticles as well; for example, in the host material ZnO when doping concentration of Co increases, the dielectric constant (ε’) also increases, similarly for each sample. We already know from XRD results that increasing the Co doping level in host ZnO leads to larger crystallites. The dielectric constant (ε’) of nanoparticles increases as crystallite size increases [35]. The distortion that occurs at the lattice structure when nearby Zn atoms are replaced by additional Co atoms is what causes the increase in dielectric constant (ε’) [36].

#### 3.5.2 Imaginary part of dielectric constant (ε’’)

**Fig 8:**
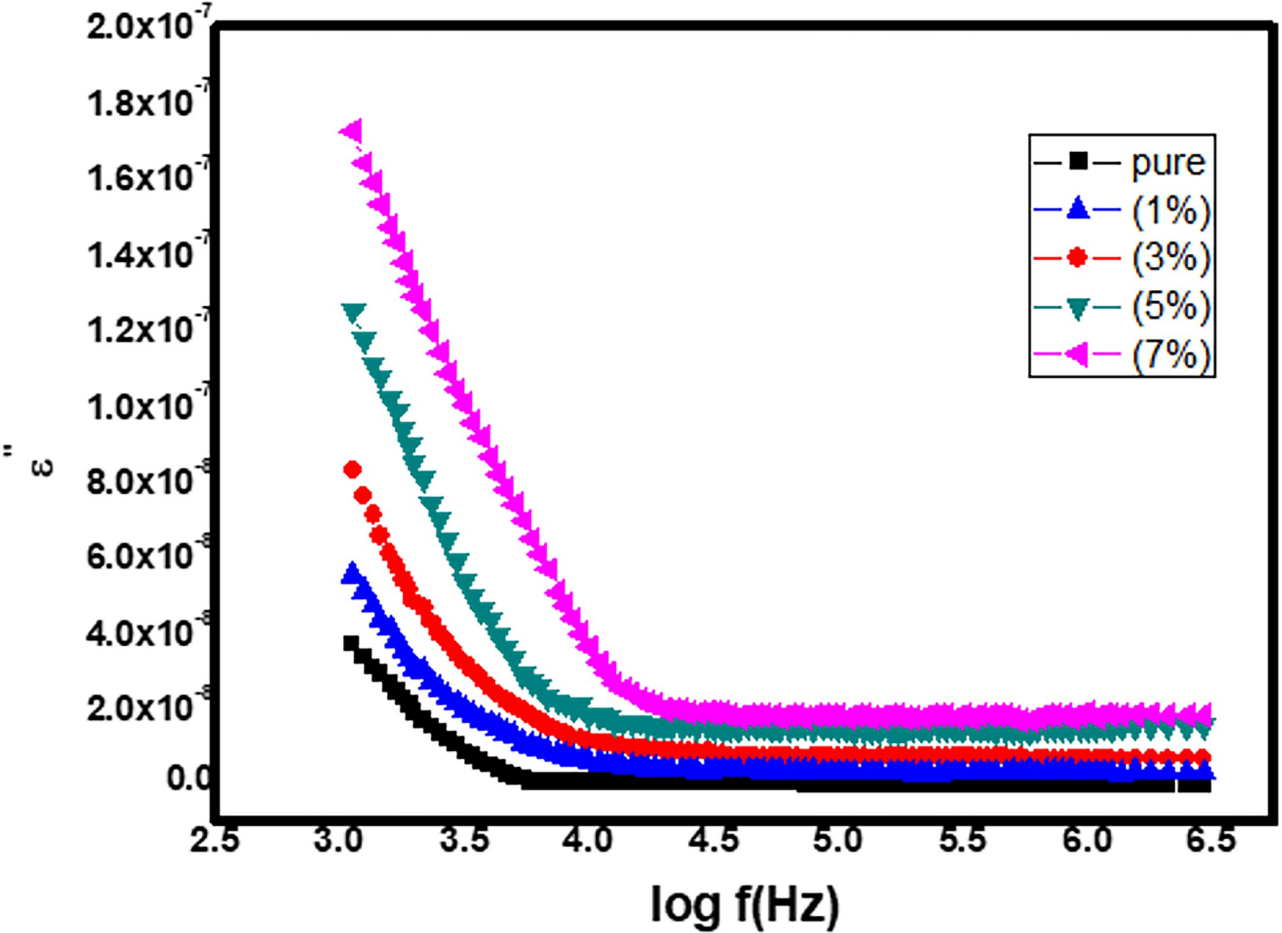
Show imaginary part of dielectric constant with varying frequency.

The imaginary component of dielectric constant’s (ε’’) is shown in Fig 8 as a function of log frequency for artificial Zn_1-x_Co_x_O nanoparticles with x = 0.00, 0.01, 0.03, 0.05, and 0.07. All synthesized nanoparticles show up on the graph with a large dielectric constant (ε’’) at low frequencies and vice versa, just as we saw with the real component of dielectric (ε’), something that has been elucidated by the Maxwell Wagner model and Koop’s theory. The inset graph in Fig 8 shows how the amount of Co^2+^ doped organized nanoparticles affect the imaginary portion of the dielectric constant (ε’’). As the applied frequency is increased, the phenomenon of polarization diminishes, and at higher frequencies, the dipoles exhibit autonomous behavior on the applied frequency, as seen in Fig 8. The inset graph in Fig 8 shows that the imaginary component of the dielectric constant (ε’’) increases at a similar rate to the increase in Co^2+^ doping content. Since neighboring Zn^2+^ atoms were swapped out for Co^2+^ atoms, the lattice structure has been distorted, leading to a rise in the imaginary portion of the dielectric constant (ε’).

#### 3.5.3 AC conductivity

By using the following equation, the ac conductivity of undoped and Co-doped ZnO-NPs has been calculated.


$$\sigma_{ac}=\varepsilon '\varepsilon \circ \omega \;tan\delta$$ (5)

**Fig 9:**
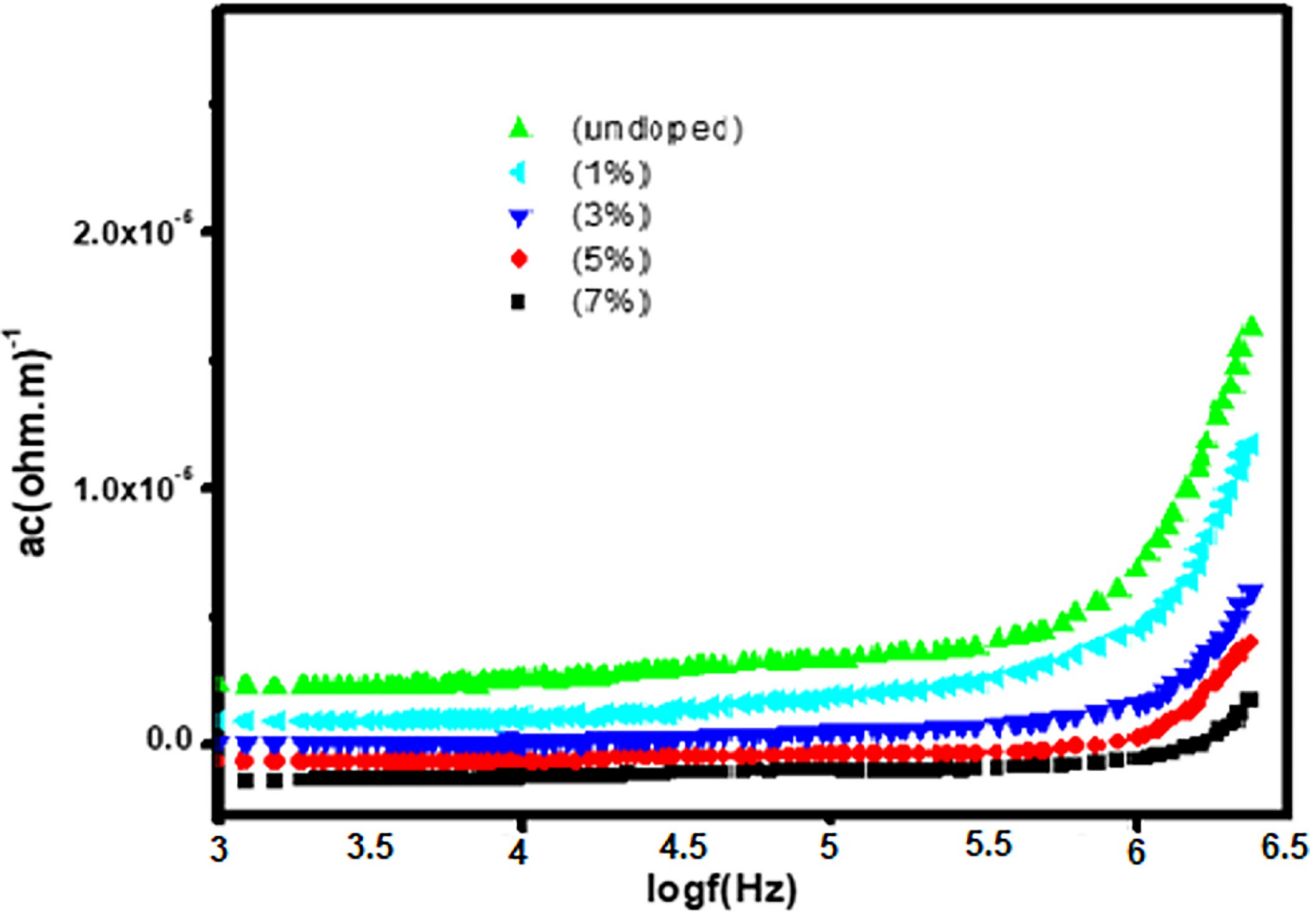
a.c conductivity of pure and Co-doped ZnO (Zn_1-x_Co_x_O) nanoparticles.

The term σ_ac_ in the equation above stands for the a.c conductivity of NPs, ε∘ represents the free-space permittivity (8.85×10^−12^ Fm^-1^), applied field frequency and dispersion factor is denoted by ω and tanδ, ε^’^ for the real portion of the dielectric. It is clear from Fig 9 that the frequency with which the a.c. conductivity (σ_ac_) increases. The increment in a.c. conductivity (σ_ac_) is due to acceleration of the electron hopping process. The graph also shows that a.c conductivity (σ_ac_) decreases as Co^2+^ dopant content in ZnO increases because Co^2+^ dopant content causes faults in the lattice of ZnO, which may cause the grains to separate at the grain boundaries. As a result, as the level of doping increases, so do the number of faults. This enabled grain boundaries flaw barriers, which in turn caused a roadblock of charge carriers and results in dropping down the a.c conductivity of the sample.

## 4. Conclusions

Pure and Co-doped ZnO-NPs were prepared using a simple and cost-effective Co-precipitation route. XRD and SEM were used for structural and morphological study. There is no evidence of any additional phases in the X-ray diffraction (XRD) graphs of pure and doped ZnO-NPs with different concentrations of Co (X = 0.00, 0.01, 0.03, 0.05, and 0.07). The average crystallite size, which spans from 29 to 33.5 nm, was calculated using Scherrer’s equation. SEM micrographs show that pure and Co-doped ZnO (Zn_1-x_Co_x_O) are spherical in shape. By using tauc plots the band gap of pure and Co-doped ZnO-NPs was calculated, a reduction in band gap is observed (i.e., 3.4 eV to 2.7 eV) by increasing Co^2+^ ratio in host material. According to the dielectric graphs, the real portion of the ε’ and the imaginary part of the ε’’ are largest at small frequency and nominal at high frequency. The dielectric constant of all Co-doped ZnO-NPs continuously enhanced from 4.0 × 10^−9^ to 2.25 × 10^−8^, and the dielectric loss reduced from 4.0 × 10^−8^ to 1.7 × 10^−7^ when Co content increased from 0.01 to 0.07%, whereas a.c. conductivity increases with increasingly applied frequency. Alternating current’s conductivity increases exponentially when frequency is applied. Results suggests that Co doping with various concentrations significantly affects the dielectric properties of ZnO-NPs. The prepared particles have a wide and promising applications in UV photodetector, optoelectronic and spintronic.
